# Causal relationship between telomere length and osteonecrosis: Bidirectional two-sample Mendelian randomization analysis

**DOI:** 10.1097/MD.0000000000039324

**Published:** 2024-08-16

**Authors:** Hao Liu, Wei Yan, Jinsong Li, Di Luo, Dezhi Yan

**Affiliations:** a The First Clinical College of Medicine, Shandong University of Traditional Chinese Medicine, Jinan, Shandong Province, China; b Department of Orthopedic Joints, Affiliated Hospital of Shandong University of Traditional Chinese Medicine, Jinan, Shandong Province, China.

**Keywords:** causality, genome-wide association studies, Mendelian randomization, osteonecrosis, single nucleotide polymorphisms, telomere length

## Abstract

Recent mounting evidence suggests that shortening of telomere length (TL) is associated with impaired bone health; yet, a genetic causal relationship between TL and osteonecrosis remains uncertain. This study aimed to investigate the potential causal relationship between TL and osteonecrosis using bidirectional two-sample Mendelian randomization (MR). Genome-wide association study summary statistics for TL were sourced from the IEU Open genome-wide association study project, while osteonecrosis data were obtained from the FinnGen Biobank database. A range of MR methodologies—including inverse variance weighting, MR-Egger, weighted median, simple mode, and weighted mode—were utilized for analysis, along with the MR-Egger intercept method for horizontal pleiotropy assessment, and Cochran Q and leave-one-out methods for heterogeneity testing. The forward MR analysis indicated a significant causal relationship between TL and osteonecrosis, suggesting that genetically predicted shorter TL is associated with an elevated risk of developing osteonecrosis (OR = 0.611, 95% confidence interval 0.394–0.948, *P *= .028). The reverse MR analysis revealed no significant influence of osteonecrosis on TL (OR = 0.999, 95% confidence interval 0.994–1.005, *P* = .802). Analyses for heterogeneity and horizontal pleiotropy yielded robust results. Our study demonstrates that individuals with shorter TL have an increased risk of developing osteonecrosis, whereas osteonecrosis has no effect on TL.

## 1. Introduction

Osteonecrosis, also termed avascular necrosis, represents a multifactorial condition classified primarily into 2 categories: traumatic and nontraumatic.^[1]^ The principal causes of nontraumatic osteonecrosis include glucocorticoid usage and alcohol abuse, with impaired bone homeostasis playing a pivotal role in its pathogenesis.^[2]^ Osteonecrosis predominantly occurs between the ages of 30 and 60 years and typically leads to a severe loss of function due to the poor mechanical strength of the necrotic bone tissue.^[3,4]^ Consequently, identifying the etiology and risk factors for osteonecrosis, as well as exploring possible pathophysiological mechanisms, is essential for early diagnosis, treatment, and improvement of quality of life.

Telomere is a TTAGGGGG nucleotide repeat sequence located at the chromosome termini where it binds to specific protein complexes, collectively maintain the integrity of the genome.^[5,6]^ Numerous evidence suggests that genomic changes resulting from environmental factors and the epigenome can affect skeletal health.^[7–12]^ Telomeres, essential for DNA protection and genomic stability, have been shown to be strongly associated with articular cartilage aging and osteoporosis.^[13,14]^ Wong et al also revealed a potential association between telomere shortening, telomere dysfunction, and impaired bone health,^[15]^ and therefore, a causal relationship between telomere shortening and impaired bone homeostasis can be hypothesized. To date, the genetic research focused on telomere length (TL) in osteonecrosis has been limited, and causal investigations into the relationship between TL and the development of osteonecrosis could illuminate novel aspects of its pathogenesis.

Mendelian randomization (MR) analysis is increasingly being employed to deduce the impact of exposure factors on outcomes.^[16]^ This method utilizes genetic variants as instrumental variables (IVs) to establish causation. Given that confounders typically do not correlate with genetic variants, observable differences in outcomes between individuals with and without the variant can be ascribed to differences in exposure. Observational studies have inherent susceptibilities to confounding variables and reverse causation. In contrast, MR analysis serves to effectively mitigate these biases, offering more compelling evidence than is typically afforded by traditional observational studies.^[17]^ To date, no research employing MR methods has been undertaken to investigate the relationship between TL and osteonecrosis. Consequently, we performed a bidirectional two-sample MR study to explore the potential causal association between TL and osteonecrosis, thereby advancing our understanding of the disease mechanisms underlying osteonecrosis.

## 2. Materials and methods

### 2.1. Study design

**Figure 1.**
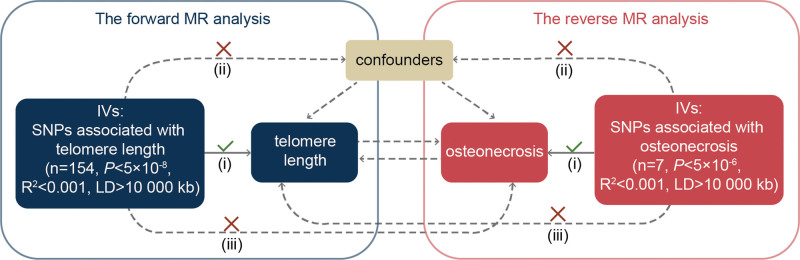
Schematic representation of bidirectional two-sample Mendelian randomization hypothesis.

To explore potential causal associations between exposure and related outcome, MR analysis will use single nucleotide polymorphisms (SNPs) that are closely associated with exposure as IVs. SNPs used as IVs must satisfy 3 assumptions^[18]^: (i) IVs must be strongly related to exposure factors; (ii) IVs must not impact outcomes through pathways other than exposure; and (iii) IVs must not be directly associated with outcomes unless such an association is established through exposure factors. The flow of the bidirectional MR design is depicted in Figure 1.

### 2.2. Data sources

Summary statistics of genome-wide association studies (GWAS) for TL were sourced from the IEU OPEN GWAS project (https://gwas.mrcieu.ac.uk/), encompassing 472,174 samples. Osteonecrosis data, derived from a GWAS published in 2021 in the FinnGen Biobank database (https://www.finngen.fi/en), encompassed a cohort of 210,179 participants, including 604 individuals with osteonecrosis and 209,575 controls, across 16,380,447 SNPs. The aforementioned populations are European, mitigating potential bias arising from population stratification. Table 1 presents detailed information.

**Table 1:** GWAS information on telomere length and osteonecrosis.

Exposure/outcome	GWAS ID	Author	Year	Sample size	Population	Number of SNPs
Telomere length	ieu-b-4879	Codd	2021	472,174	European	20,134,421
Osteonecrosis	finn-b-M13_OSTEONECROSIS	–	2021	392,580	European	16,380,447

GWAS = genome-wide association study, SNP = single-nucleotide polymorphism.

### 2.3. Selection of instrumental variables

In this study, SNPs related to TL were identified using *P* < 5 × 10^-8^ as the threshold for significance. For a broader statistical inference, the significance threshold for osteonecrosis-associated SNPs in reverse MR analysis was established at *P *< 5 × 10^-6^. SNPs with an r^2^ ≥ 0.001 and within 10,000 kb linkage disequilibrium distance from other SNPs were excluded by calculating the paired-chain imbalance.^[19]^ Phenoscanner platform (http://www.phenoscanner.medschl.cam.ac.uk/) was utilized to identify SNPs directly linked to the outcome factors. *F*-statistics were derived using the formula [(r^2^ × (n-^2^))/(1 ‐ r^2^)], with only IVs that had an *F*-statistic exceeding 10 chosen to mitigate the bias from weak IVs.^[20]^ The remaining SNPs constituted the final IVs for exposure factors.

### 2.4. MR analysis

Our study utilized 5 approaches to ascertain the causal influence of TL on osteonecrosis, namely: inverse variance weighting (IVW), MR Egger, weighted median, simple mode, and weighted mode. Utilizing the IVW method as the primary analysis, it amalgamates the Wald estimates of each SNP, considering them as natural experiments, and mimics a randomized controlled trial through the principle of random segregation, enforcing a zero intercept in the regression slope. This may induce bias in the presence of an invalid IV.^[21,22]^ To affirm the robustness of our findings, supplementary analyses were conducted employing the 4 additional methods. A *P*-value <.05 indicated a causal connection between the exposure factor and the outcome.

### 2.5. Sensitivity analysis

Heterogeneity among discrete genetic variants was assessed using Cochran Q test statistic, and a *P*-value >.05 implied an absence of heterogeneity across causal analyses.^[23]^ The MR-Egger intercept test, along with MR-PRESSO, was deployed to identify and adjust for horizontal pleiotropy through the exclusion of outliers.^[24,25]^ Funnel plots served to evaluate the potential bias within the analyzed results. Leave-one-out analyses were executed to gauge the influence of individual SNPs on the MR findings.

Statistical analyses were conducted using R software, version 4.1.2, with the “TwoSampleMR” and “MR-PRESSO” packages. A *P*-value of <.05 was deemed to indicate statistical significance.

## 3. Results

### 3.1. Influence of telomere length on osteonecrosis

**Figure 2.**
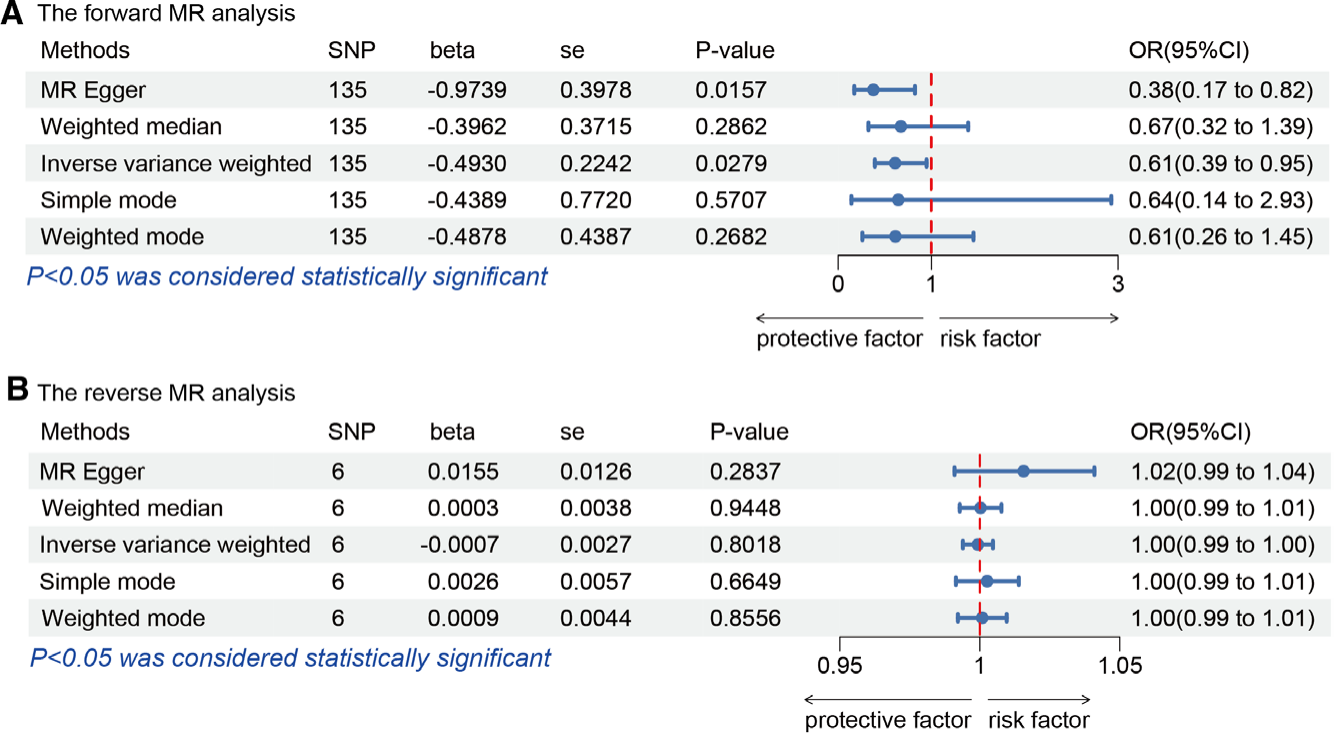
Bidirectional MR analysis results. MR = Mendelian randomization.

**Figure 3.**
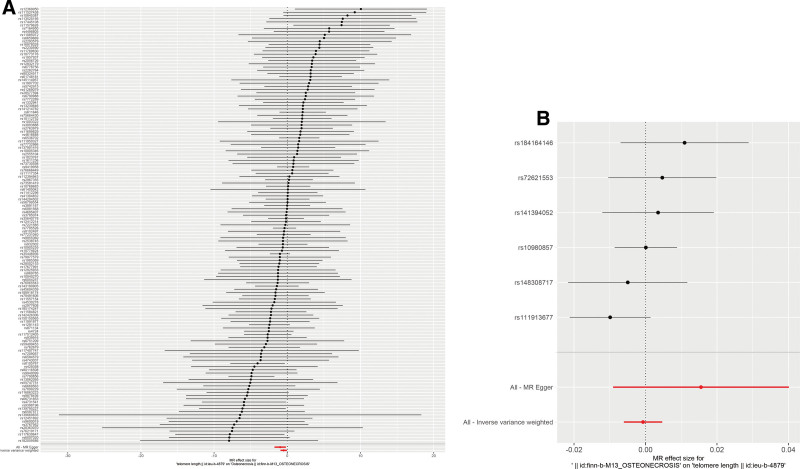
Forest plot of bidirectional MR analysis results. (A) Forest plot of forward MR analysis; (B) forest plot of reverse MR analysis. MR = Mendelian randomization.

**Figure 4.**
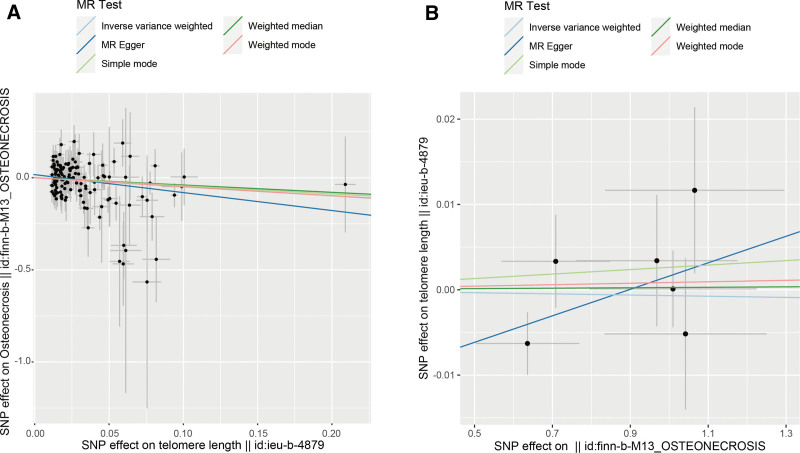
Scatter plots of bidirectional MR analysis results. (A) Scatter plots of forward MR analysis; (B) scatter plots of reverse MR analysis. MR = Mendelian randomization.

**Figure 5.**
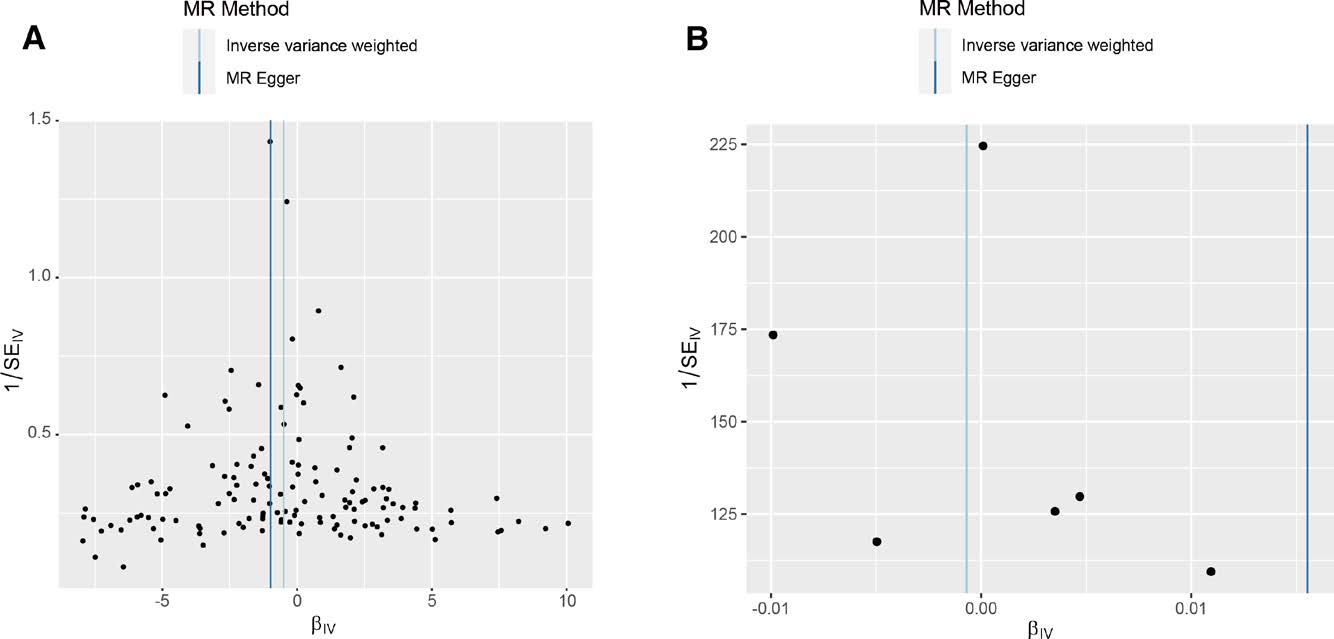
Funnel plots of bidirectional MR analysis. (A) Forward MR analysis funnel plot; (B) reverse MR analysis funnel plot. MR = Mendelian randomization.

**Figure 6.**
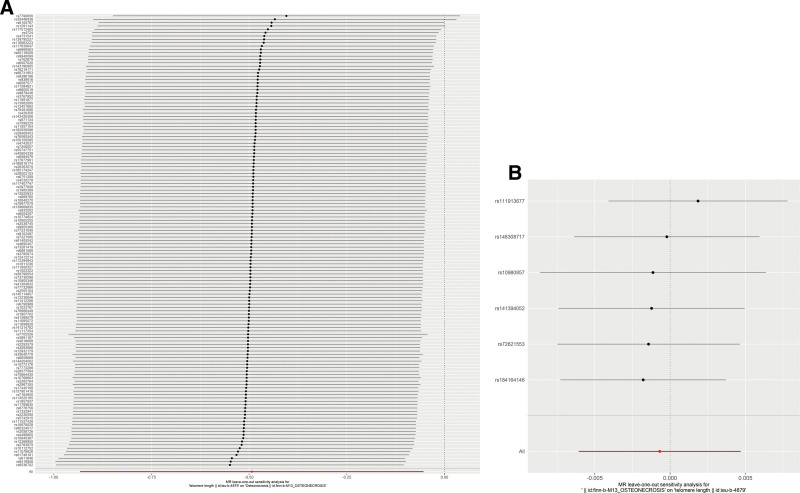
Leave-one-out analysis forest plot. (A) Forward MR analysis; (B) reverse MR analysis. MR = Mendelian randomization.

Following screening, 154 independent SNPs linked to TL were identified, with the lowest F-statistic for these IVs exceeding 10, aligning with the hypothesis that weak instrument bias is unlikely to confound the causal inference. Meanwhile, the MR-Egger intercept revealed an absence of horizontal pleiotropy (intercept = 0.017, *P* = .146), detailed in Table 2, and MR analysis utilizing the 5 methodologies are presented in Figure 2A. Overall, the primary IVW analysis indicated that genetically predicted TL has a causal association with osteonecrosis (OR = 0.611, 95% confidence interval [*CI*] 0.394–0.948, *P* = .028). The MR-Egger results (OR = 0.378, 95% *CI* 0.173–0.824, *P *= .016) reinforced the conclusion. Additionally, consistent results were derived from the weighted mode, weighted median and simple mode analyses (Fig. 2A). Forest plots and scatter plots illustrating the relationship between osteonecrosis and TL are depicted in Figures 3A and 4A, respectively. The Cochran Q test, applied through MR-Egger and IVW methods, indicated an absence of heterogeneity among the SNPs (Table 3). Funnel plots suggested a reduced likelihood of confounding effects on causality (Fig. 5A). Leave-one-out analysis revealed that the sequential exclusion of TL-associated SNPs did not significantly alter the analysis results (Fig. 6A).

**Table 2:** MR-Egger test for horizontal pleiotropy.

	Exposure	Outcome	Intercept	SE	*P*-value
Forward MR analysis	Telomere length	Osteonecrosis	0.017	0.011	.146
Reverse MR analysis	Osteonecrosis	Telomere length	‐0.014	0.011	.257

**Table 3:** Heterogeneity test.

Exposure	Outcome	MR Egger		Inverse variance weighted		MR-PRESSO	
		Q	*P*-value	Q	*P*-value	Global test *P*	Distortion test *P*
Telomere length	Osteonecrosis	131.878	.511	134.019	.483	0.585	NA
Osteonecrosis	Telomere length	3.471	.482	5.221	.390	0.354	NA

### 3.2. Influence of osteonecrosis on telomere length

Initial screening pinpointed 7 independent SNPs linked to osteonecrosis, each with an F-statistic exceeding 10, supporting the “correlation” assumption. The MR-Egger intercept exhibited no indication of horizontal pleiotropy (intercept = ‐0.014, *P* = .257) (Table 2), and Cochran Q test detected no heterogeneity (Table 3). IVW analysis indicated no causal link between osteonecrosis and TL (OR = 0.999, 95% *CI* 0.994–1.005, *P* = .802), with corroborative results from additional methods presented in Figure 2B. Forest plots and scatter plots illustrating the relationship between osteonecrosis and TL are depicted in Figures 3B and 4B, respectively. The funnel plot is presented in Figure 5B. Leave-one-out analysis indicated that the sequential exclusion of osteonecrosis-linked SNPs did not significantly alter the analytical outcomes (Fig. 6B).

## 4. Discussion

This study is the first to evaluate the causal association between TL and osteonecrosis using multiple complementary MR methods. The forward MR analysis suggested that a genetic inclination towards shorter TL is associated with a heightened risk of osteonecrosis. Conversely, reverse MR analysis revealed no evidence of a causal link between osteonecrosis and TL.

Recent studies on TL in the context of skeletal-related diseases have highlighted its potential as a diagnostic and prognostic marker,^[26]^ with growing evidence suggesting telomere shortening may contribute to the pathogenesis of these diseases via epigenetic mechanisms. For instance, Zhai et al measured TL in patients with hand osteoarthritis and controls, finding shorter telomeres in the osteoarthritis group.^[27]^ Tamayo et al also reported an association between shorter TL and osteoporosis.^[28]^ Although numerous studies have focused on osteonecrosis surgery, there remains a deficiency in molecular machine.^[29,30]^ Lee et al^[31]^ analyzed TL in synovial tissue from patients with osteonecrosis of the femoral head (ONFH) and femoral neck fractures, finding no TL differences between the groups. Subsequent studies revealed an association between elevated erythrocyte sedimentation rates and TL shortening, which may imply that ONFH may be associated with an inflammatory process triggered by telomere shortening. While ONFH is the most prevalent form of osteonecrosis, it does not fully represent the potential relationships between other types like talar and humeral head necrosis with TL. Presently, research supporting a correlation between TL and various forms of osteonecrosis is still insufficient. Furthermore, aforementioned studies may be biased due to factors such as small sample sizes, small age of the covered population, age distribution disparities, and alcohol use. And TL is not only determined by environmental factors such as increased oxidative stress and chronic inflammation, but also by genetic factors.^[32]^ TL varies among individuals at birth; therefore, it may be difficult to elucidate the causal relationship between TL and osteonecrosis without considering genetic factors. In this study, the causal relationship between TL and osteonecrosis was confirmed using MR methods, and sensitivity analysis and the reverse MR analysis reduced the potential confounding and minimized reverse causation, thereby strengthening the final causal inference.

Although the precise mechanisms underpinning osteonecrosis remain unclear, diminished osteogenic capacity of bone marrow mesenchymal stem cells (BMSCs), oxidative stress inhibiting osteogenesis and angiogenesis, and immunological factors are all potential contributors to the disease. TL shortening along with telomerase activity, play pivotal roles in regulating the biological functions of BMSCs and osteoblast, potentially contributing to the development of osteonecrosis. For instance, Saeed et al discovered that telomerase deficiency-induced TL shortening hastens senescence in human BMSCs in vitro; conversely, upregulating telomerase results in longer telomeres, lifespan extension, and bolstered osteogenesis in these cells.^[33]^ TL shortening is also a critical inducer of tissue inflammation, greatly affecting bone turnover. Under the influence of pro-inflammatory cytokines, the pro-inflammatory bone microenvironment enhances bone resorption and promotes apoptosis.^[33–36]^ It has been reported that TL shortening both mediates osteoblast dysfunction and increases the inflammatory microenvironment within the skeleton, thereby enhances the production of osteoclasts, leading to dysregulated bone remodeling.^[15,33]^ Furthermore, in autoimmune diseases, telomere dysfunction is dependent on the Neutrophil extracellular traps (NETs) mechanism of cathelicidin LL37 to promote small vessel vasculitis,^[37]^ which is closely associated with the development of osteonecrosis. This study revealed that TL shortening may be a potential pathway driving osteonecrosis development, underscoring its significance in disease pathogenesis, which also implies that TL is expected to be a potential indicator for monitoring and preventing osteonecrosis. Future research is necessary to delineate the precise mechanisms by which TL shortening affects osteonecrosis.

Despite the MR analysis yielding robust and reliable findings, certain limitations were present: (1) the sample size for outcome factors was limited, necessitating studies with larger sample sizes to corroborate these conclusions; (2) while controlling for race minimized racial bias, it also resulted in limited applicability of the findings to other racial groups; (3) a lack of data on osteonecrosis types and severity meant that the relationship between TL and these factors remained unevaluated; (4) genetic variation accounts for merely one aspect influencing exposure changes, with the impact of environmental, lifestyle, and epigenetic factors requiring additional investigation. As a result, these findings offer only a partial explanation of the causal effect of TL on osteonecrosis.

In conclusion, our study indicates that individuals with shorter TL have an increased risk of developing osteonecrosis, while osteonecrosis does not significantly alter TL. These findings indicate that TL shortening may be a potential pathway that drives the development of osteonecrosis and offer valuable insights for clarifying the possible mechanisms behind the disease.

## Acknowledgments

The authors thank the reviewer for editing the manuscript. This work was supported by the Shandong Provincial Natural Science Foundation Joint Special Fund Project (grant number ZR2021LZY002) and the 2020 Qilu Outstanding Young Talent Cultivation Project for Health and Wellness (grant number rc2021002-23).

## Author contributions

**Conceptualization:** Hao Liu, Di Luo.

**Formal analysis:** Hao Liu.

**Funding acquisition:** Di Luo.

**Investigation:** Dezhi Yan.

**Writing – original draft:** Hao Liu.

**Writing – review & editing:** Wei Yan, Jinsong Li.
